# Creating a Foundation for the Visualization of Intracranial Cerebrospinal Fluid Using Photon-Counting Technology in Spectral Imaging for Cranial CT

**DOI:** 10.3390/diagnostics14222551

**Published:** 2024-11-14

**Authors:** Anna Klempka, Philipp Neumayer, Alexander Schröder, Eduardo Ackermann, Svetlana Hetjens, Sven Clausen, Christoph Groden

**Affiliations:** 1 Department of Neuroradiology, University Medical Centre Mannheim, Medical Faculty Mannheim, University of Heidelberg, Theodor-Kutzer-Ufer 1-3, 68167 Mannheim, Germany; 2 Department of Medical Statistics and Biomathematics, Medical Faculty Mannheim, University of Heidelberg, 68167 Mannheim, Germany; 3 Department of Radiation Oncology, University Medical Centre Mannheim, Medical Faculty Mannheim, University of Heidelberg, 68167 Mannheim, Germany

**Keywords:** photon-counting CT, intravascular imaging, intracranial CSF, spectral imaging, non-enhanced imaging

## Abstract

Background: Recent advancements in computed tomography (CT), notably in photon-counting CT (PCCT), are revolutionizing the medical imaging field. PCCT’s spectral imaging can better visualize tissues based on their material properties. This research aims to establish a fundamental approach for the in vivo visualization of intracranial cerebrospinal fluid (CSF) using PCCT. Methods: PCCT was integrated to distinguish the CSF within the intracranial space with spectral imaging. In this study, we analyzed monoenergetic +67 keV reconstructions alongside virtual non-contrast and iodine phase images. This approach facilitated the assessment of the spectral characteristics of CSF in patients who did not present with intra-axial pathology or inflamation. Results: Our findings illustrate PCCT’s effectiveness in providing distinct and clear visualizations of intracranial CSF structures, building a foundation. The signal-to-noise ratio was quantified across all measurements, to check in image quality. Conclusions: PCCT serves as a robust, non-invasive platform for the detailed visualization of intracranial CSF. This technology is promising in enhancing diagnostic accuracy through different conditions.

## 1. Introduction

Photon-counting computed tomography (PCCT) creates new possibilities by enabling accessible spectral imaging without additional radiation exposure. PCCT is emerging as a groundbreaking tool in neuroradiology, significantly enhancing our ability to visualize the brain and other complex structures, as well as identifying pathologies throughout the body [1,2]. Further research is essential to build the foundation for utilizing not only the imaging capabilities of PCCT but also its spectral parameters to explore new diagnostic opportunities especially in the neuroradiology field.

This innovative technology differs fundamentally from traditional computer tomography (CT) systems by using photon-counting detectors, which count individual X-ray photons and measure their energy [3]. This seemingly small technical difference offers a range of substantial benefits, including sharper images, improved tissue differentiation, and the potential for reduced radiation doses—crucial in the delicate task of imaging the central nervous system and adjacent structures [4,5].

One of the most exciting aspects of PCCT is its capacity for spectral imaging. Unlike conventional CT scans that average the energy of incoming X-ray photons, PCCT distinguishes between photons of varying energies, allowing for more precise differentiation between tissue types. In neuroradiology, where detecting subtle variations in brain tissue can mean the difference between early diagnosis and missed conditions, this capability could be transformative [6]. For example, PCCT can distinguish between similar-looking tissues, such as calcium deposits and iodine-based contrast agents, or detect minor differences in tissue density that may signal the early stages of disease [7].

Additionally, PCCT offers a major improvement in spatial resolution, which is crucial for neuroimaging. The brain’s anatomy is highly intricate, with structures like cranial nerves and small blood vessels requiring high-resolution imaging to be seen clearly [8]. Traditional CT systems, constrained by older detector technologies, may struggle to capture these details accurately. However, photon-counting detectors can be made smaller, resulting in higher-resolution images that provide clearer views of these critical structures. Radiation exposure is always a concern in the medical imaging field, particularly in sensitive areas such as the brain and in vulnerable populations like children. PCCT’s efficient photon detection allows it to produce high-quality images while using lower radiation doses than traditional CT, even those with advanced detectors [9]. This reduction in radiation exposure enhances the safety and accessibility of CT scans, especially for repeated use when monitoring conditions over time.

In summary, PCCT is not just an incremental advancement in imaging technology—it represents a significant leap forward that has the potential to reshape the entire field of neuroimaging.

The cerebrospinal fluid (CSF) is a crucial component of the central nervous system (CNS), playing an essential role in maintaining the brain’s structural and functional integrity. This clear, colorless fluid circulates through the brain’s ventricles and the subarachnoid space surrounding the brain and spinal cord [10]. The CSF serves several vital functions, including protecting the brain from mechanical shocks, maintaining a stable environment for neural activity, and facilitating the exchange of nutrients and waste products between the brain and the bloodstream [11,12]. One of the primary roles of CSF is to act as a protective cushion for the brain. Given the brain’s soft, gelatinous nature and its confinement within the rigid skull, any sudden impact could cause significant injury if not for the buffering effect of CSF, which absorbs and dissipates mechanical forces. This cushioning function is essential for preserving the brain’s intricate neural networks and ensuring the continuity of cognitive and sensory functions, which can be compromised by issues such as the CSF leaks [13,14].

Beyond its protective role, the CSF is key in maintaining homeostasis within the central nervous system (CNS). The brain depends on a tightly regulated environment for optimal function, and the CSF helps regulate this environment by exchanging ions, nutrients, and metabolic waste. It distributes critical substances like glucose and electrolytes necessary for neuronal function while also removing waste generated by neural metabolism, preventing the buildup of harmful substances that can indicate disease [15,16].

The CSF also provides buoyancy to the brain, effectively reducing its apparent weight within the skull. This buoyancy decreases pressure on brain structures, particularly blood vessels and nerve roots, helping to prevent tissue deformation and ensuring the free flow of blood that delivers oxygen and nutrients essential for brain function [17].

In the context of medical research, the CSF is of significant interest due to its accessibility and the wealth of information it provides about the internal environment of the central nervous system. Studying the CSF can offer valuable insights into the fundamental processes that regulate brain function, such as intracranial pressure, fluid dynamics, and the biochemical exchange between the brain and the bloodstream [18]. These insights have been instrumental in understanding pathological conditions like cranial hypertension, subarachnoid bleeding, and meningitis [19].

The spectral imaging of CSF using PCCT represents a cutting-edge, non-invasive approach to assessing the physiological status of the CNS. PCCT’s ability to capture spectral data by distinguishing the energy levels of individual X-ray photons can offer a significant enhancement over traditional CT techniques. This capability could allow for a more nuanced analysis of the CSF, enabling the detection of subtle changes in its composition that reflect the health of the brain and spinal cord.

As noted, the CSF plays a critical role in maintaining homeostasis in the CNS. Traditionally, analyzing the CSF has required invasive lumbar punctures to collect samples for biochemical examination. However, our study aims to lay the groundwork for using PCCT to image the normal CSF spectrally. This approach could lead to future developments where clinicians are able to assess the CSF’s composition and dynamics non-invasively, following a PCCT scan. Such imaging could be particularly useful for monitoring over time, where repeated invasive procedures are impractical or pose significant risks.

In conclusion, the spectral imaging of CSF using PCCT could offer a powerful, non-invasive tool for monitoring the physiological state of the CNS. By harnessing the advanced capabilities of photon-counting technology, further research is needed to build a foundation for understanding the CSF’s spectral composition and establishing diagnostic norms, similar to those used in the current laboratory parameters.

## 2. Materials and Methods

### 2.1. Patients

The institutional review board approved this retrospective study. We reviewed non-contrast (non-enhanced) cranial CT scans performed using a PCCT scanner from 1 January 2024 to 23 December 2021. This study included patients who met the following criteria: no intra-axial pathology, no history of intra-axial surgeries, no implanted intracranial foreign objects, no recent bleeding history, no acute inflammation of any kind, and no intracranial hypertension. From the patients who underwent scans with this PCCT scanner, we selected 92 cranial CT scans from 77 individuals. Due to brain edema and very slim ventricles, two patients were excluded, leaving 75 individuals. The participants had a median age of 76.5 years (standard deviation: ±13.25), comprising 25 women and 50 men. This group of patients were also partially presented in our other previous study [20].

### 2.2. Image Acquisition

In this study, all cranial CT scans were performed using the Naeotom Alpha PC CT scanner from Siemens Healthineers (Forchheim, Germany). The scanning protocol was standardized, featuring a 120 kV setting with a quality reference of 72 mAs, an ME67 filter, a pitch factor of 0.35, a rotation time of 0.5 s, and a matrix size of 512 × 512. The images were acquired using spiral acquisition. This is a typical clinically used cranial protocol for PCCT in our institution. Each participant consistently underwent this cranial CT protocol, ensuring that the radiation parameters were kept constant to achieve uniform imaging quality and reproducibility. Spectral imaging data sets were consistently included in the process.

### 2.3. Evaluation

**Figure 1:**
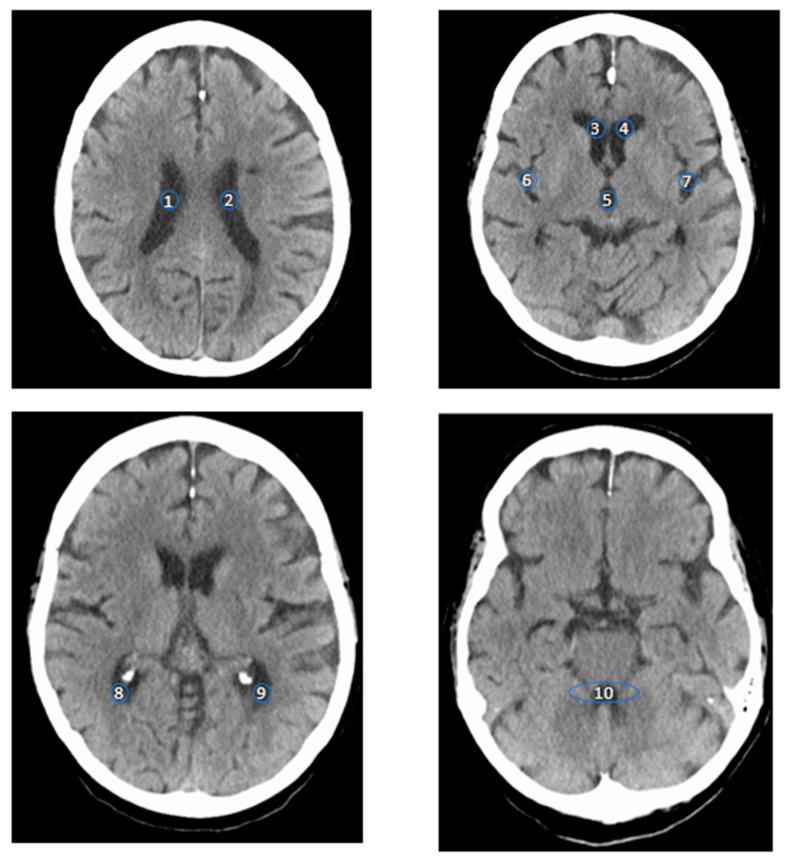
The four images show axial CT scans of the brain at different axial levels. The numbers indicate where the measurements were conducted, as described below: (1) the central part of the right lateral ventricle; (2) central part of the left lateral ventricle; (3) anterior horn of the right lateral ventricle; (4) anterior horn of the left lateral ventricle; (5) third ventricle (III); (6) right sylvian fissure; (7) left sylvian fissure; (8) posterior horn of the right lateral ventricle; (9) posterior horn of the left lateral ventricle; and (10) fourth ventricle (IV).

Measurements were taken over an area of roughly 15 mm^2^ as a region of interest (ROI) at the following locations: the lateral ventricles, including each of the anterior horn, central part, and posterior horn, on both sides separately; the fourth ventricle; the third ventricle; and the Sylvian fissure at the level of the insula, on both sides separately (Figure 1). The ROI size was determined based on previous studies by other authors [8] and a phantom study [21] to maximize the signal-to-noise ratio, while also aligning with the measured anatomically accessible areas.

Baseline Hounsfield Unit (HU) values and standard deviations (SD) were obtained from monoenergetic reconstructed data at +67 keV for each ROI, covering both the virtual native and iodine phases. These measurements were performed with a 1 mm slice thickness using a soft kernel reconstruction. The images were analyzed using Siemens’ syngo.via software, version 8.3.

### 2.4. Sigal-to-Noise Ratio

We assessed image quality by calculating the signal-to-noise ratio (SNR), which is defined as the ratio of the target’s signal intensity to the background noise in the image. To determine the SNR, the average signal intensity in Hounsfield Units (HU) was divided by the standard deviation (SD) of measurements obtained from monoenergetic images at a +67 keV setting.

### 2.5. Statistical Analysis

All statistical analyses were conducted using the SAS software version 9.4 (SAS Institute Inc., Cary, NC, USA). For variables that were approximately normally distributed, the results are expressed as mean values along with their standard deviations. In the case of non-normally distributed data, the median, minimum, and maximum values are reported.

## 3. Results

The focus of our study is on percentiles, as the density of CSF can vary within a narrow range. We observed relatively homogeneous results for the internal CSF spaces across all measured areas, as shown in Table 1. However, the results for external CSF spaces show some other variations, as detailed below:

Monoenergetic 67 keV HU Measurements: The measurements of the internal CSF spaces varied slightly. The range between the 10th and 90th percentiles shows that the 10th percentile values range between 6 and 7 HU, while the 90th percentile values range between 12 and 14 HU. The highest value recorded was 16 HU, and the lowest was 5 HU. Measurements of the external CSF spaces (Sylvian fissure) presented slightly higher values, with the 10th percentile ranging between 8 and 9 HU, the 90th percentile ranging between 17 and 18 HU, and the 95th percentile reaching 19 HU, while the 5th percentile was between 5 and 8 HU; see Table 1a.

Virtual Non-Contrast (VNC) Phase: The measurements in the VNC phase between the 10th and 90th percentiles were slightly lower, ranging from 3.5–5 HU to 12–15.5 HU for the internal CSF spaces. For the external CSF spaces, the range was between 6 and 18.5 HU. The 95th percentile reached 18 HU, while the 5th percentile was between 0 and 4 HU for internal spaces and 4–5 HU for external spaces, with a maximal HU of 21; see Table 1b.

Iodine Phase: For internal ventricles, the measurements consistently showed negative values, ranging from −2 to −4 HU at the 10th percentile and 5 to 7 HU at the 90th percentile. The external CSF spaces demonstrated similar, yet more homogeneous, values ranging from −3 to −4.5 HU at the 10th percentile and 7 HU at the 90th percentile. The 95th percentile reached 9 HU, while the 5th percentile was between −3 and −6 HU for internal spaces and −5 HU for external spaces, with a maximal HU of 10; see Table 1c.

Signal-to-Noise Ratio (SNR): The mean SNR for all CSF measurements in internal spaces ranged from 2.20 to 8.17, with the highest value observed in the left lateral ventricle posterior horn. In the external CSF spaces, the mean SNR ranged from 3.40 to 3.34; see Table 1d.

## 4. Discussion

The aim of this study was to characterize the physiological status of CSF using spectral imaging with the intention of establishing normative values for our patient cohort. By defining these values, we aim to propose them as potential benchmarks for routine CSF testing, which could aid in the early detection of pathological conditions. However, it is important to acknowledge that a significant limitation of our study is the absence of laborchemical CSF analysis from healthy individuals and the slightly old patients’ group. It is important to acknowledge that our study did not investigate differences across age or sex groups, and limited literature addresses this topic through imaging-specific analyses, and further research could provide valuable insights [22]. Including such data would have provided a more comprehensive understanding of normal CSF physiology, thus enhancing the applicability and robustness of our findings.

Our study involved measurements taken from multiple locations within the CSF spaces, which yielded two distinct sets of data that must be analyzed separately. First, due to the inherent dynamics of the CSF flow and absorption [23], we anticipated some variability in flow rates across different regions. This variability could potentially lead to differences in the measurements, either due to the flow rate itself or because of subtle variations in fluid density within the brain’s fluid compartments.

An important aspect to consider is SNR measurement, particularly the observation that the highest signal intensity was recorded in the lateral ventricles, specifically in the anterior horn and lateral regions. This finding strengthens the hypothesis that signal intensity may be influenced by the CSF dynamics or the patient’s positioning within the epicenter of the measurement of PCCT scanner.

Moreover, it is noteworthy that measurements within the Sylvian fissure also demonstrated high values. In this region, the CSF flow is expected to differ from that observed in the ventricles due to variations in the processes of CSF absorption, production, and circulation [24,25]. This distinction may provide further insight into the relationship between the CSF flow and signal intensity. The CSF is composed primarily of water, along with electrolytes, proteins, and other solutes [26]. In conventional CT imaging, it appears as hypodense spaces. In certain pathological conditions, such as subarachnoid hemorrhage or inflammation, the CSF may exhibit hyperattenuation on CT, either diffusely or in localized regions, often observed as sediment within the dorsal horns of the ventricles, while patients are lying on their back for examination [27]. The pathological status of CSF inflammation is a serious condition that often cannot be diagnosed with sufficient accuracy using conventional CT imaging in the majority of cases. In certain situations, as described above, some characteristics in CT imaging may provide some diagnostic clues. However, these findings are often subtle and, on their own, insufficient to establish a definitive diagnosis. They are frequently confirmed and preceded by an evident clinical presentation or cerebrospinal fluid analysis [28].

It is noteworthy that all measurements in the monoenergetic 67 keV imaging and virtual non-contrast phase consistently showed positive values, whereas the 5th and 10th percentiles of the iodine phase included negative values. This observation may suggest a resemblance between the CSF and saline or water, as demonstrated in some phantom studies of spectral imaging [29]. In this context, spectral imaging and advanced techniques like PCCT hold promise for enhancing diagnostic accuracy. The ability to detect subtle differences in tissue composition and fluid characteristics through these advanced imaging methods could potentially be leveraged to develop algorithms or diagnostic patterns as others research already shown [30,31]. Such patterns could be tested and validated through randomized studies, offering the possibility of distinguishing between normal and pathological conditions in a way that conventional CT cannot.

One of the key challenges in advancing this approach lies in the need for in vivo studies involving patients with conditions such as meningitis. By comparing the spectral imaging results of patients with pathological CSF to those with healthy CSF, researchers could develop a clearer understanding of the diagnostic potential of this technology. Currently, the data available on this topic are limited, and more research is needed to fully explore the potential benefits of spectral imaging in the diagnosis of CSF-related pathologies. The development of such an approach could revolutionize the diagnostic process for neurological conditions, particularly those involving the central nervous system. For instance, in cases of meningitis, early and accurate diagnosis is crucial for effective treatment. Traditional imaging techniques often fail to provide the level of detail necessary to identify the condition in its early stages. PCCT spectral imaging, on the other hand, could offer a more detailed view, allowing for earlier intervention and better patient outcomes.

CSF examination, in clinical practice, requires obtaining the CSF via a lumbar puncture, which can be burdensome for patients. This procedure poses particular risks for patients with coagulation disorders, as it may be associated with a risk of bleeding. Additionally, some patients may decline providing consent for such an invasive procedure. Other side effects of lumbar puncture included emergence of fistulas, infection, or nerve damage [32]. Replacing this intervention with a non-invasive imaging-based evaluation of the cranial region would represent a revolutionary advancement. However, the current state of research in this area is still in its infancy. The hypothesis that spectral imaging can significantly improve the diagnosis of CSF-related conditions needs to be rigorously tested through clinical trials and studies involving a larger and more diverse patient population. These studies should aim to establish baseline measurements for both healthy and pathological CSF, which would serve as a reference for future diagnostic efforts. Additionally, exploring the correlation between spectral imaging findings and clinical outcomes could provide valuable insights into the clinical relevance of this technique.

In conclusion, while the concept of using spectral imaging and PCCT enhance the diagnosis of CSF-related conditions is promising, it remains a largely theoretical approach at this stage. Further research, particularly involving in vivo studies of patients with confirmed conditions like meningitis, is necessary to validate this approach. If successful, this line of inquiry could represent a significant advancement in the field of diagnostic imaging, leading to more precise and early detection of serious neurological conditions.

## 5. Conclusions

Lastly, while our study provides valuable initial data on the physiological status of CSF as assessed by spectral imaging, the limitations highlight the need for further research. Addressing these limitations will be crucial for fully understanding the potential of this technique in clinical diagnostics.

## Figures and Tables

**Table 1:** The following tables (a–d) present the measurement results for different parts of the ventricular system, including the Sylvian fissure. Abbreviations: Right LV AH—right lateral ventricle anterior horn; Right LV CP—right lateral ventricle central part; Right LV PH—right lateral ventricle posterior horn; Left LV AH—left lateral ventricle anterior horn; Left LV CP—left lateral ventricle central part; Left LV PH—left lateral ventricle posterior horn; 3rd V—3rd ventricle; 4th V—4th ventricle; and SF—Sylvian fissure L (left) R (right).

a. Monoenergetic +67 keV HU—Measurements (HU)				
	5th Percentile	10th Percentile	90th Percentile	95th Percentile
Right LV AH	5	6	14	15
Right LV CP	5	6	12	13
Right LV PH	6	7	14	16
Left LV AH	6	6.5	13	15
Left LV CP	5	6	12	13
Left LV PC	6	7	14	15
3rd V	5	6	14	15
4th V	6	6	13,5	15
SF R	8	9	17	19
SF L	5	8	18	19
**b. Virtual Native Phase (HU)**				
	**5th Percentile**	**10th Percentile**	**90th Percentile**	**95th Percentile**
Right LV AH	3	5	13.5	15
Right LV CP	2	3	13	14
Right LV PH	2	4	15.5	18
Left LV AH	4	5	14	17
Left LV CP	2	3.5	12	14
Left LV PC	0	3	13	14
3rd V	2	3	14.5	16
4th V	3	4	12	14
SF R	4	6	18.5	21
SF L	5	6	18	19
**c. Iodine Phase (HU)**				
	**5th Percentile**	**10th Percentile**	**90th Percentile**	**95th Percentile**
Right LV AH	−5	−4	5	7
Right LV CP	−5	−3	6	7
Right LV PH	−6	−4	6,5	7
Left LV AH	−5	−4	6	7
Left LV CP	−4	−3	5	6
Left LV PC	−4	−2	7	9
3rd V	−5	−3	6	7
4th V	−3	−2	7	7
SF R	−5	−4.5	7	10
SF L	−5	−3	7	8
**d. SNR**				
	**SNR**			
Right LV AH	2.39			
Right LV CP	2.29			
Right LV PH	2.58			
Left LV AH	8.17			
Left LV CP	2.20			
Left LV PC	2.65			
3rd V	2.33			
4th V	2.36			
SF R	3.34			
SF L	3.20			

## Data Availability

The data presented in this study are available upon reasonable request from the corresponding author.
